# Analytical performance evaluation of different test systems on serum creatinine assay

**DOI:** 10.1002/jcla.24206

**Published:** 2021-12-26

**Authors:** Lina He, Jinqi Yu, Guang Han, Di Huang, Liqiao Han, Qiaoxuan Zhang, Yunxiu Wang, Zemin Wan, Xian‐Zhang Huang, Yujuan Xiong, Xiaobin Wu

**Affiliations:** ^1^ The Second Clinical Medical College Guangzhou University of Chinese Medicine Guangzhou China; ^2^ Department of Laboratory Medicine The Second Affiliated Hospital of Guangzhou University of Chinese Medicine Guangzhou China; ^3^ Department of Laboratory Medicine Panyu Hospital of Chinese Medicine Guangzhou University of Chinese Medicine Guangzhou China

**Keywords:** analytical performance, creatinine assay, interference, method comparison, test system

## Abstract

**Background:**

Serum creatinine (SCr) is a useful diagnostic marker for the assessment of renal function. Accurate quantitation of SCr is clinically important in calculation of glomerular filtration rate (GFR).

**Method:**

To confirm whether there are differences in SCr between enzymatic kits of different manufacturers, the analytical performance of the matched and open test system in the measurement of SCr was evaluated. The analytical performance evaluation was conducted according to the Clinical and Laboratory Standards Institute (CLSI) guidelines. Precision, accuracy, linearity, dilution, lower limit of measurement and analytical interference were studied between the two test systems.

**Results:**

The performance of SCr from the open test system was in compliance with the matched test system with good precision, accuracy, and linearity. In presence of most common interferents, both test systems could lead to accurate creatinine results except for the existence of specified drugs. For dobutamine, the open test system showed better anti‐interference performance than the matched system.

**Conclusion:**

This study provides referable opinions for clinical laboratory selection on the test system and a framework for future analogous studies based on different test systems.

## Graphical abstract

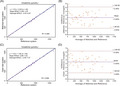

## 1 INTRODUCTION

Creatinine is a breakdown product of creatine phosphate from muscle and protein metabolism.^1^ It is produced and released by the body at a constant rate into the blood, and it is then carried to the kidneys through the blood circulation.^2^ In the body's daily metabolism, creatinine is generated and subsequently excreted in the urine because it can pass through the glomerular membrane but rarely be absorbed in the renal tubules.^3^, ^4^

In clinical practice, the level of serum creatinine (SCr) is generally used as a significant indicator for renal function evaluation.^3^ SCr determination is a widely applied diagnostic test to evaluate glomerular filtration rate (GFR) that when renal function is impaired, the creatinine level rises.^5^

Clinical methods for serum creatinine determination include chemical and enzymatic methods. Chemical methods are susceptible to the interference of certain creatinine derivatives or homologues, resulting in inaccurate SCr level.^2^ Although the chemical method has been modified, compensation is still needed to ensure accurate SCr determination results.^6^ In comparison, the application of the enzymatic method is more expensive in SCr quantification, but it avoids the poor specificity of the Jaffe method and has strong anti‐interference ability as well as less reagent toxicity.^6^ Therefore, the enzymatic method is a cost‐effective approach for SCr determination, which is routinely employed by most clinical laboratories.

An accurate creatinine determination is pivotal; therefore, analytical performances should be concerned, as suggested by NKDEP.^7^ Numerous diagnostic reagent kits with manufacturer‐claimed performances can be chosen in clinical laboratory; however, results may have large variations between different test systems. In this study, we hope to compare the performance of the two test systems in order to select a system with better performance in clinical practice, so as to provide clinicians with accurate results.

A matched test system means the analyzer, reagent kit, and calibrators are from the same manufacturer.^8^ Similarly, an open test system refers to a system where reagent and calibrator of the same manufacturer can be adapted to distinct analyzers, so that users could make choice on what reagent kits and calibrators as they want on open test systems.^9^ This study established a performance evaluation method for the application of open reagents of creatinine on the Roche Cobas 8000 C702 Chemical Analyzer. The Shino‐Test SCr quantification kit and the Roche Cobas 8000 C702 Chemistry Analyzer constituted an open test system. Under the condition of eliminating the different influences of cuvettes, light sources, detectors, etc., evaluations were conducted between an open test system and a clinically used matching test system (Roche SCr quantification kit on Roche Cobas 8000 C702 chemical analyzer). Performances (including precision, linear relationship, and accuracy), consistency (method comparison), and the influence of common interfering substances were compared and evaluated.

The results showed that the open test system presented a similar efficiency in performance evaluation with the matched test system. It should be noted that when evaluating the influence of dobutamine, a medication used in the treatment of cardiogenic shock and severe heart failure,^10^ the open test system had better anti‐interference ability. Therefore, this study discussed the differences in performances between the two test systems. Consequently, either the open test system or the matched test system can meet the needs of clinical testing on creatinine. If dobutamine is present when measuring the creatinine level, we recommend choosing the Shino‐Test detection system to determine the creatinine concentration.

## 2 MATERIALS AND METHODS

### 2.1 Analyzer, reagents, and other material

In our study, the open test system namely the Shino‐Test creatinine reagent kit (Lot. R‐I #C842,R‐II #C842, Shino‐Test Corp., Tokyo, Japan) and Roche Cobas 8000 c702 chemistry analyzer (Roche Diagnostics, Mannheim, Germany) was applied to determine creatinine, according to the manufacturer's instructions. The determination results were analyzed and compared with those obtained from the matched test system (Roche creatinine reagent kit (Lot. #310314) and the same analyzer). All system setup, calibration, and QC testing of the analyzer had been finished prior to sample analysis. The test systems were operated according to the manufacturer's instructions and the laboratory's standard procedures. To evaluate the precision, two levels of quality control materials (PreciControlClinChem Multi 1, PCCC1, Lot. #144 514–02; PreciControlClinChem Multi 2, PCCC2, Lot. #144 527–04) from Roche Diagnostics (Mannheim, Germany) and another two levels of quality control materials (Liquid Assayed Multiqual Control 695,696; Lot. #45752, 45753) from Bio‐Rad Laboratories (CA, USA) were utilized throughout the study. Accuracy analyses were partially carried out on a Waters ACQUITY UPLC^®^ system in positive electrospray ionization mode, namely the reference system (isotope‐dilution liquid chromatography/tandem mass spectrometry, ID‐LC/MS method) in our study. Calibration and QC testing of the reference system had been qualified prior to sample analysis. For linearity evaluation on samples of high‐level creatinine, standard reference material of creatinine (product # SRM 914a, National Institute of Standards and Technology) was applied. For evaluation of analytical specificity, interference substances were used by mixing with serum pool at three concentration levels and subsequently measured creatinine level of the mixture. In the study, the influences of hemolysis, hyperbilirubinemia, lipemia, and drugs were evaluated. Interference substances include purified bilirubin (product #B4126, Sigma Chemical Company, St Louis, MO, USA), fat emulsion (20% Intralipid; Sino‐Swed Pharmaceutical Corp., Wuxi, China), fat‐soluble vitamin (Vitalipid N; 10 ml/ampoule, Sino‐Swed Pharmaceutical Corp., Wuxi, China), vitamin C (ascorbic acid; 250 mg/ml, CSPC Ouyi Pharmaceutical, Shijiazhuang, China), dobutamine (10 mg/ml, Zhejiang Ruixin Pharmaceutical, Zhejiang, China), and calcium dobesilate (2, 5‐dihydroxybenzene sulfonate; 500 mg/capsule, Xi'an Lijun Pharmaceutical, Xi'an, China).

### 2.2 Samples

Serum samples were obtained by collecting surplus samples from routine creatinine tests at the Clinical Laboratory of Guangdong Provincial Hospital of Chinese Medicine. The study was reviewed and approved by the Research and Ethics committee of our institution, and all participants signed their consents prior to the study. Lipemic and hemolyzed samples were excluded. Specimens for method comparison study were collected from left‐over clinical patient samples (*N* = 34), with creatinine levels ranging from 45 to 1610 μmol/L. Serum samples were all collected from adults, and the gender is randomly distributed who had not taken medication mentioned in this study. Samples at different creatinine levels were collected as well in accuracy evaluation study and subsequently blended at different ratios to reprepare specimens at 45 levels to be assigned by the reference method. Specimens at each level was divided into three aliquots. In addition, samples with creatinine levels close to the medicine decision levels (MDLs) were collected and applied in linearity and interference evaluation.

### 2.3 Precision

The evaluation of precision was carried out using four quality control materials according to the EP15‐A2 evaluation protocol of the Clinical and Laboratory Standards Institute (CLSI).^11^ QC materials were divided into five aliquots per level and frozen at −80 ºC. One aliquot of each level was thawed at room temperature 30 minutes before analysis and gently inversed to homogenize. Triplicate creatinine measurements of each level were then daily performed for a total of five nonconsecutive days (*N* = 15 per level). Results were presented in terms of coefficient of variation (CV%). The calculation is performed using the formulas as shown in the previous study.^12^ A recommended minimum CV for serum creatinine analysis is less than 3.2%, which is three quarters the intraindividual biological variation.^13^

### 2.4 Accuracy

In the study of accuracy evaluation, five EQA materials were measured in triplicate by both test systems, and the test results were compared within a medically allowable bias to NCCL‐given (NCCL, National Center for Clinical Laboratories) target values. The accuracy was accepted if they were within target values ±1/2 TEa% (allowable total error; 6%). Furthermore, forty‐five fresh frozen/thawn patient samples evenly distributed over the measuring interval were determined in triplicate by the reference system and the two test systems. Results from the two test systems were compared to those from the reference system by performing a Passing‐Bablok regression, and the estimated values at the MDLs were then calculated. The relative biases between estimated values and MDLs were compared with the allowable specification. In addition, a Bland‐Altman (BA) plot of percent differences of the two test systems and IDMS results were constructed.

### 2.5 Linearity, dilution, and lower limit of measurement

The test for linearity was carried out in accordance with the CLSI protocol. We used standard reference material of creatinine to simulate high‐level creatinine which claimed by manufacturers. The creatinine concentration of the median samples covered high concentration of samples that can be seen in clinical practice and the reference interval range. Samples with the level of creatinine close to the upper limit of reference interval were applied to evaluate the linearity of low‐level creatinine. The linearity of high‐level, median‐level, and low‐level creatinine were evaluated using eleven respective pools. In the linearity of high‐level creatinine, besides the pool containing the highest‐level concentration, the other ten pools were from mixtures of the mentioned serum sample with saline solution at ratios of 0:10, 1:9, 2:8, 3:7, 4:6, 5:5, 6:4, 7:3, 8:2, 9:1, and 10:0. In the linearity of low‐level creatinine, besides the pool containing creatinine concentration that close to the upper limit of reference interval, the other pools were prepared accordingly. Besides the pool containing creatinine concentration that close to either the lower limit of reference interval or clinical samples with high‐level creatinine, the other nine pools were prepared correspondingly. Each pool was measured in triplicate. The linearity results were presented as the equation from the linear regression including slope, Y intercept, and the correlation coefficient (*R* ^2^). As acceptance criteria, the slope should be close to 1 and *R* ^2^ ≥ 0.99.

The dilution study was conducted by measuring clinical samples diluted by saline solution, and the allowable dilution ratio was acceptable when the bias was within 6%.

Isotonic saline solution (0.9%) was used to evaluate the lower limit of measurement. Each specimen was tested in 20 replicates, and mean values and standard deviation (SD) were then calculated. Lower limit of measurement was defined as the mean value +3 SD according to the manufacturer.

### 2.6 Method comparison

Comparison study was performed to compare the open test system to the matched test system, according to the CLSI EP09‐A3 guidelines,^14^ and subsequently, the correlation was evaluated. A total of 34 patient serum samples were aliquoted into two fractions, which were measured using the two systems within 2 hours. A Passing‐Bablok regression was then conducted based on measured values, and relative bias at MDLs was calculated. A Bland‐Altman (BA) plot was made accordingly.

### 2.7 Analytical interferences

Interference study was carried out by adding different solutions of interferents to serum pools at three levels of creatinine. Each sample was determined in triplicate using the two test systems. The values of creatinine level of original pool were set as baseline values in which the measurement values were compared with in order to calculate the percentage creatinine recovery. Significant interference was defined when a recovery change exceeded 10% of the baseline values.^15^, ^16^, ^17^ The relative bias of each specimen was calculated from the observed value and the baseline value. Table and plots were constructed to illustrate the influence of common interferents.

#### 2.7.1 Interference of hemolysis, hyperbilirubinemia, and lipemia

To study the influence of hemolysis, hemolysate was added to each serum pool aliquot according to Fleming and Swaminathan.^18^ The hemolysate was prepared from EDTA‐anticoagulated whole blood. The blood was centrifuged to separate plasma and cells, and the cells were then washed three times with saline solution (0.9% NaCl). Later, the supernatant was removed and distilled water was added to the cells. After 15 min standing at 4°C, the mixture was centrifuged and separated. Subsequently, the concentration of hemoglobin for the hemolysate was determined at 60 g/L. A specified volume of hemolysate was then spiked with different serum pool aliquots at 1:9 ratio to obtain interference samples with hemoglobin concentration at 6 g/L. The other dilutions were prepared by serially diluted with the previous samples and saline at proper ratio to final concentrations of hemoglobin at 4.8, 3.6, 2.4, and 1.2 g/L. For the assessment of interference for bilirubin, the study was conducted by dissolving 20 mg of purified bilirubin to prepare the interferent pool at 200 mg/dl (3420 mmol/L) and the pool was then consecutively diluted with serum pool aliquots to obtain samples with 68.4, 136.8, 205.2, 273.6, and 342 mmol/L bilirubin. The study of potential interference of lipemia was performed according to Glick.^19^ Generally, serial fat emulsion or fat‐soluble vitamin dilutions were prepared following instructions and added to serum pool aliquots to obtain interference samples with 1.3, 5.1, 9.2, 13.0, and 16.6 mmol/L fat emulsion or 1.3, 5.5, 9.6, 13.3, 15.5, and 15.9 mmol/L fat‐soluble vitamin.

#### 2.7.2 Interference of drugs

Interference studies of vitamin C or dobutamine were carried out by mixing serum pool aliquots with serial dilutions of vitamin C solution or dobutamine injection. Final target concentrations were 0.2, 0.4, 0.6, 0.8, and 1.0 mg/ml vitamin C as well as 4, 8, 12, 16, and 20 μg/ml dobutamine,respectively. Additionally, the influence of another drug, calcium dobesilate, was also evaluated. Calcium dobesilate powder in capsule was dissolved in saline and spiked into serum pool aliquots to prepare interferent samples containing 2, 4, 8, 16, 32, and 64 mg/L calcium dobesilate.

### 2.8 Statistical analysis

Statistical analysis was performed using the Microsoft Excel (Microsoft Corporation, USA), GraphPad Prism software (GraphPad Software, CA, USA), and R Statistical Software (version 4.0; R Foundation for Statistical Computing, Vienna, Austria). The linear regression analysis was conducted following the Passing‐Bablok method. The measurement results of method comparison and accuracy study were compared using the Bland‐Altman analysis.

## 3 RESULTS

### 3.1 Precision

The open test system yielded within‐run CVs ranging from 0.31% to 0.80% and total CVs ranging from 0.29% to 1.19%, while within‐run CVs and total CVs of the matched test system were 0.56%‐0.95% and 1.00%‐1.20%, respectively (Table 1). The precision evaluation results showed acceptable coefficients of variation within 1/2 allowable total error (TEa%).

**TABLE 1:** Precision evaluation by the two test systems

Measurand	*N*	Matched test system			Open test system		
		Mean (μmol/L)	CV_within_%	CV_total_%	Mean (μmol/L)	CV_within_%	CV_total_%
PC1	15	90.4	0.95%	1.20%	112.07	0.76%	1.19%
PC2	15	357.87	0.76%	1.00%	404.87	0.40%	0.62%
B2	15	174.8	0.57%	1.15%	177.2	0.80%	0.87%
B3	15	816.47	0.56%	1.16%	825.2	0.31%	0.29%

Note

The mean creatinine levels represented 15 replicate measurements of each test system on four quality control material over 5 days.

Abbreviations: CV_total_%, total coefficient of variation; CV_within_%, within‐run coefficient of variation; *N*, number of measurements.

### 3.2 Accuracy

**FIGURE 1:**
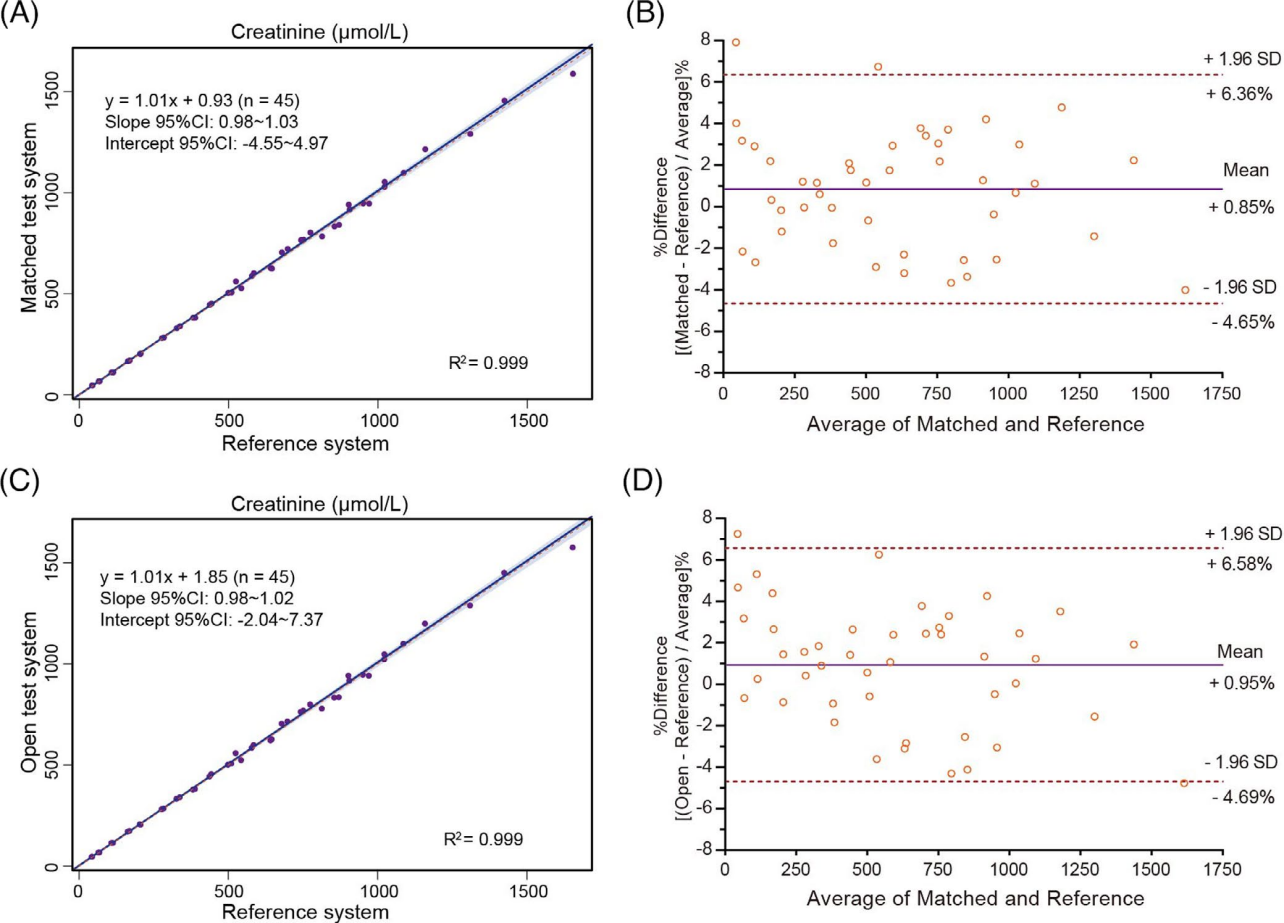
Comparison between the two test systems and the reference system using the Passing‐Bablok (PB) regression analysis and Bland‐Altman (BA) plot. The creatinine values were determined in 45 samples with systems above. (A) Comparison of the matched test system and reference system by PB. (B) Comparison of the matched test system and reference system by BA. (C) Comparison of the open test system and reference system by PB. (D) Comparison of the open test system and reference system by BA. The light purple area in the Passing‐Bablok analyses shows the 95% confidence interval; the orange dotted lines represent the identity line (X = Y); the purple solid lines are regression lines. The solid lines in the Bland‐Altman plots show the mean percent difference, and the dotted lines display the mean percent difference ±1.96 SD

Both test systems showed high accuracy. Firstly, the relative biases against NCCL target values of either test system were within ±6% limit, which were clinically acceptable (Table 2). Moreover, the creatinine levels of 45 samples measured by two test systems were compared with the values assigned by the IDMS method. The Passing‐Bablok regression fit is Y = 1.006 (95% confidence interval (CI):0.981 to 1.021) X + 1.849 (95% CI: −2.043 to 7.366) for the open test system with correlation coefficient (r) over 0.99, indicating that the open test system obtained a good correlation with the reference system (ID‐LC/MS method) on the whole range of measure (Table 3). According to the Bland‐Altman analysis, the percent differences were small on creatinine determination, suggesting that the two observed test systems and the reference system reached good agreement (Figure 1). These data indicated that the two observed test systems could lead to accurate determination results for patient samples on clinical practice.

**TABLE 2:** Accuracy of creatinine values determined by the two test systems

Sample number	Creatinine, μmol/L			Relative bias, %	
	Target value	Matched test system	Open test system	Matched test system	Open test system
20180201	125	125.0	124.0	0.0	−0.8
20180202	407	407.3	409.0	0.1	0.5
20180203	330	330.0	330.3	0.0	0.1
20180204	604	603.7	609.0	−0.1	0.8
20180205	235	234.7	236.3	−0.1	0.6

Note

The NCCL EQA acceptance limit of ±6% was considered clinical allowable.

**TABLE 3:** Accuracy analysis by comparing with the reference system

Test System	*N*	*R* ^2^	Slope (95% CI)	*y* Intercept (95% CI)	MDL (μmol/L)	Estimate value (μmol/L)	Absolute bias	Relative bias (%)	Allowable bias (%)
Matched	45	0.999	1.01 (0.98–1.03)	0.93 (−4.55 to 4.97)	53	54.41	1.41	2.66	6
					141	143.20	2.20	1.56	
					530	535.70	5.70	1.08	
Open	45	0.999	1.01 (0.98–1.02)	1.85 (−2.04 to 7.37)	53	55.17	2.17	4.09	6
					141	143.70	2.70	1.91	
					530	535.03	5.03	0.95	

Note

The estimate values at MDLs are calculated based on the Passing‐Bablok regression fit.

Abbreviations: MDL, medical decision level; *N*, number of measurements; *R* ^2^, correlation coefficient.

### 3.3 Linearity, dilution, and lower limit of measurement

The results of the linearity and dilution studies were shown in Table 4 and Table 5, respectively. The linear regression analysis of respective high, median, and low level for each of the two test systems showed a line with a good correlation coefficient (*R* ^2^ > 0.99) over the range tested. All biases were <6% for the matched and open test system, when the dilution ratio was below 4 and below 10, respectively. The lower limits of measurement were 2.06 and 2.46 μmol/L for the matched and open test system, respectively, indicating comparable performance (data not shown).

**TABLE 4:** Evaluation of the linearity of creatinine at different levels

Test system	Theoretic value of creatinine level, μmol/L	95% CI		*R* ^2^	Regression fit
		Slope	Intercept		
Matched					
Low	0.0–131.7	0.966–0.973	0.59–1.13	0.999	*Y* = 0.97*x* + 0.86
Medium	35.0–1370.0	0.97–1.00	1.28–24.67	0.999	*Y* = 0.99*x* + 12.97
High	0.0–8949.0	0.89–0.94	26.76–265.20	0.999	*Y* = 0.92*x* + 145.98
Open					
Low	0.0–128.8	0.98–1.01	−0.88 – 1.19	0.999	*Y* = 1.00*x* + 0.15
Medium	34.3–1360.3	0.98–1.00	−0.36 – 20.34	0.999	*Y* = 0.99*x* + 9.99
High	0.0–8762.2	0.990–0.999	−20.25 – 32.55	0.999	*Y* = 0.99*x* + 6.15

Abbreviations: CI, confidence interval; *R* ^2^, correlation coefficient.

**TABLE 5:** Definition of the allowable dilution ratios

Dilution	Matched test system			Open test system		
	Theoretical, μmol/L	Measured, μmol/L	Relative bias, %	Theoretical, μmol/L	Measured, μmol/L	Relative bias, %
1	8185.7	8185.7	0.0	8755.0	8755.0	0.0
2	4092.8	4332.7	5.9	4377.5	4368.0	−0.2
4	2046.4	2119.3	3.6	2188.8	2090.3	−4.5
8	1023.2	1138.3	11.2^a^	1094.4	1107.0	1.2
10	818.6	892.5	9.0^a^	875.5	871.0	−0.5

^a^ Exceed the 6% criteria of the clinical laboratory.

### 3.4 Method comparison

**FIGURE 2:**
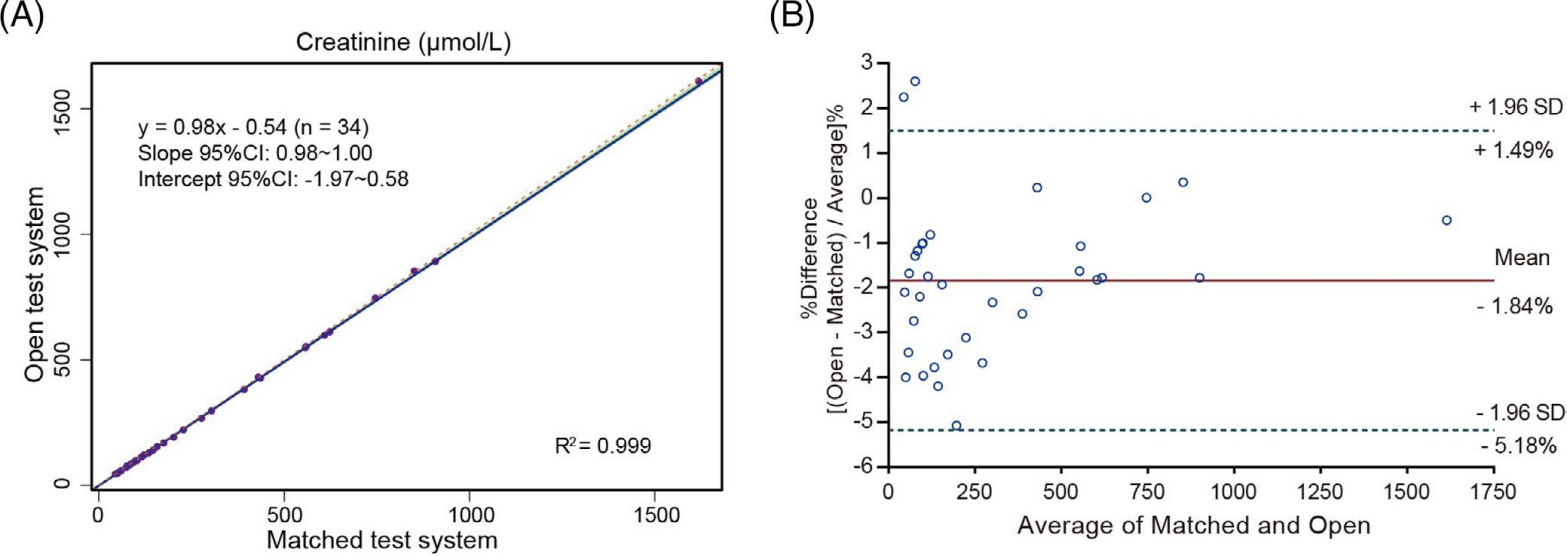
Method comparison results for creatinine (μmol/L). (A) The Passing‐Bablok regression and (B) Bland‐Altman analysis of the open test system vs matched test system. The light green area in (A) shows the 95% confidence interval; the orange dotted line represents the identity line (X = Y); the purple solid line is the regression line. The solid line in (B) shows the mean percent difference, and the dotted lines display the mean percent difference ±1.96 SD

The correlation between systems was obtained by analyzing 34 samples with creatinine level in a dynamic range from 45 to 1610 μmol/L. The matched test system was used as a comparative method. The measured values from the two systems were analyzed and described with the Passing‐Bablok regression fit: *Y* = 0.984 (95% CI: 0.977 to 0.996) *X* − 0.541 (95% CI: −1.967 to 0.580) (Figure 2A). Additionally, the relative biases at medical decision levels were −2.62%, −1.98%, and −1.70%, respectively, which were acceptable. Besides, the Bland‐Altman plot in Figure 2B showed a mean percent difference of −1.842% and 95% limits of agreement ranging from −5.178% to 1.494%, and only 2 out of 34 data points fell out of the 95% limits of agreement depicted by the upper and lower line.

### 3.5 Analytical interferences

**FIGURE 3:**
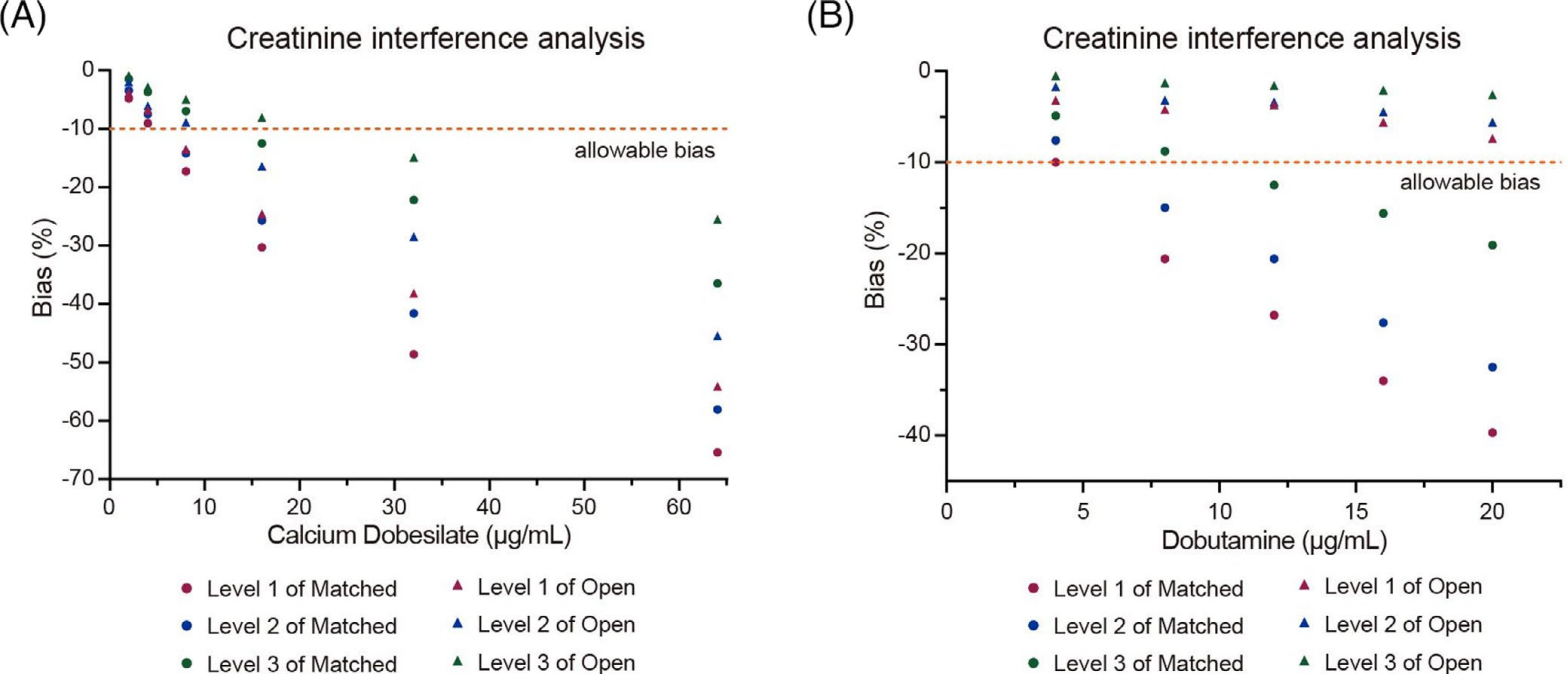
Effect of interference from calcium dobesilate and dobutamine on creatinine determination. Samples of creatinine at three different levels with interference substances or not were determined in triplicate using the two test systems, respectively. The acceptable bias against baseline values is set at 10%

The interference experiment was conducted by comparing determination results of serum samples with/without interfering substances, with an allowable bias of ±10%. As shown in Table 6, the biases caused by following interfering substances, hemoglobin (up to 6 g/L), bilirubin (up to 342 mol/L), and lipids (up to 20.8/15.5 mmol/L) were all <±10% for serum samples at different levels. In the presence of 8 μg/ml calcium dobesilate, two test systems exhibited relative biases ranging from −13.5% to −17.3% in the low‐level creatinine group (Figure 3A). Moreover, the same calcium dobesilate concentration produced significant interference in the matched test system in the medium‐level creatinine group with the bias of −14.2% (Figure 3A). The open test system exceeded the acceptable criteria of relative bias of ±10% in the medium‐level creatinine group at calcium dobesilate concentration of 16 μg/ml (Figure 3A). In the elevated‐level creatinine group, calcium dobesilate concentration at 16 μg/ml caused significant interference (relative bias of −12.5%) in the matched test system, and 32 μg/ml calcium dobesilate negatively affected creatinine determination with relative bias of −14.9% in the open test system (Figure 3A). As shown in Figure 3B, in the presence of 8 μg/ml dobutamine, the matched test system exhibited notable negative interference in the low‐ and medium‐level creatinine groups, with relative biases of −20.6% and −15.0%, respectively. At dobutamine concentration of 12 μg/ml, a noticeable interference was also observed, with relative bias of −12.5%, which exceeded the acceptable criteria of ±10%. In contrast, no unacceptable interference was observed in the low‐, medium‐, and elevated‐level creatinine groups for the open test system when the exogenous dobutamine concentrations were up to 20 μg/ml. Considering the clinically acceptable criteria of ±10%, the open test system displayed relatively robust anti‐interference performance.

**TABLE 6:** Interference assessments for common interferents

Interferent	Relative bias from native sample					
	Matched test system			Open test system		
	Level 1	Level 2	Level 3	Level 1	Level 2	Level 3
Hemoglobin, g/L						
1.2	−4.2%	−2.0%	−1.6%	0.0%	−0.2%	−0.6%
2.4	−4.7%	−2.9%	−2.3%	0.0%	−0.7%	−0.6%
3.6	−5.1%	−2.9%	−2.0%	−1.9%	−0.4%	−0.4%
4.8	−5.6%	−2.9%	−3.4%	1.4%	0.0%	−0.6%
6.0	−5.1%	−2.7%	−3.7%	3.3%	0.4%	−0.3%
Bilirubin, mmol/L						
4	1.0%	0.7%	−0.4%	−0.5%	0.2%	0.1%
8	−1.5%	−0.2%	−0.6%	−2.0%	0.5%	−0.2%
12	−3.1%	0.2%	−0.1%	0.0%	0.0%	−0.4%
16	−3.1%	−2.2%	−2.5%	−2.0%	−1.5%	−1.9%
20	−2.6%	−2.9%	−2.4%	−3.0%	−2.7%	−2.6%
Intralipid, mmol/L						
1.3	0.0%	0.0%	0.0%	0.0%	0.0%	0.0%
5.1	0.0%	−0.2%	0.1%	0.5%	−1.2%	−0.2%
9.2	0.5%	−0.2%	−0.1%	−1.9%	−0.7%	−0.7%
13.0	1.0%	−0.2%	0.3%	−1.4%	0.7%	−0.7%
16.6	1.0%	−0.2%	−0.7%	0.0%	−0.5%	−0.3%
20.8	0.0%	1.2%	0.5%	1.0%	−1.2%	0.1%
Vitalipid, mmol/L						
1.3	0.0%	0.0%	0.0%	0.0%	0.0%	0.0%
5.5	−1.4%	−1.3%	1.3%	−0.9%	0.4%	−0.3%
9.6	−1.9%	−1.8%	−0.5%	0.0%	0.2%	−0.7%
13.3	−3.8%	−1.5%	0.1%	−0.5%	0.7%	−1.0%
15.9	−1.9%	−0.9%	0.5%	0.0%	1.1%	−1.2%
15.5	−1.9%	−2.0%	0.4%	0.0%	1.3%	−0.2%
Vitamin C, mg/ml						
0.2	1.0%	−1.0%	−0.9%	0.0%	−0.7%	−0.3%
0.4	−1.5%	−3.2%	−1.8%	−4.0%	−0.5%	−0.6%
0.6	−3.1%	−4.2%	−2.4%	−4.0%	−1.9%	−0.9%
0.8	−3.1%	−5.4%	−3.4%	−5.5%	−2.9%	−1.3%
1.0	−2.6%	−7.1%	−4.7%	−6.0%	−1.7%	−1.2%

Note

Relative bias >±10% was considered unacceptable.

## 4 DISCUSSION

Serum creatinine is a biomarker for estimating glomerular filtration rate (eGFR) in patients and provides clinicians with an assessment of renal function.^20^, ^21^, ^22^ An accurate creatinine determination is pivotal; thus, analytical performances should be concerned. Numerous diagnostic reagent kits with manufacturer‐claimed performances are provided to be chosen in clinical laboratory; however, results may vary between different test systems. In the present study, the regent kit of creatinine and analyzer from Roche Diagnostics constitute the matched test system; the regent kit of creatinine from Shino‐Test and analyzer from Roche Diagnostics constitute the open test system. We then performed the comparative evaluation of analytical performances between the two test systems.

In the precision study, satisfactory results were obtained as the CVs were lower than the desirable goal for imprecision derived from biological variation of creatinine (≤2.2%).^7^ Results show that either matched test system or open test system presents extremely low imprecision at each level evaluated. In the accuracy study, we calculated the relative bias of creatinine values measured with the test systems against the target values of EQA materials. The results showed good accuracy of each test system since the relative biases against the NCCL targets were both within ±6%. Moreover, we performed a Passing‐Bablok regression and a Bland‐Altman analysis to see whether the test systems could yield similar results of the reference system. As shown in Figure 1 and Table 3, results of each test system were in accordance with those assigned by the reference system, and relative biases at MDLs were both clinically acceptable, indicating either test system could lead to correct results for patient samples on clinical practice. Regarding the linearity of the test systems, the results showed that the correlation coefficients were >0.99 and the slopes were both close to 1, which indicated excellent linearity over ranges of high‐, median‐, and low‐level creatinine. In the method comparison study, the Passing‐Bablok regression analysis yielded a good correlation between the two test systems for determining serum creatinine with the slope and intercept close to 1 and 0, respectively. In addition, the Bland‐Altman plot presented that the two test systems were in good agreement by giving an average relative bias of −1.84%.

The traditional picrate acid (Jaffe) method used to determine serum creatinine concentration is lack of analytical specificity as it is known to be subject to interference from certain substances.^23^, ^24^, ^25^, ^26^, ^27^ The US National Kidney Disease Education Program Laboratory Working Group promoted the use of enzymatic assay for creatinine quantification, and it has been widely implemented for routine clinical laboratory use because it could offer more specific creatinine determination with improved accuracy.^7^, ^23^ However, creatinine determination by enzymatic assay is still reported that interfered by several substances.^28^, ^29^, ^30^ In our study, we investigated the anti‐interference performance of the test systems with the presence of common interferents or specific drugs. There was no significant interference observed in the presence of interferents listed in Table 6. Furthermore, the interference of another two medications, calcium dobesilate and dobutamine were analyzed as well. Calcium dobesilate (calcium 2,5‐dihydroxybenzenesulfonate) is a well‐known vaso‐protectant and also exerts protective effect on diabetic nephropathy 13 and gentamicin‐induced acute kidney injury.^31^, ^32^ Dobutamine is a medication widely used in critical care to treat shock/hypotension and augment cardiac output.^33^ Notably, our results revealed that calcium dobesilate interfered with both matched and open system in a dose‐dependent manner at concentrations greater than 8 μg/ml. Calcium dobesilate caused a significant negative interference on creatinine determination, which may result in false kidney function evaluations in patients treated with the medication. Hence, extra precautions should be taken in creatinine determination in clinical practice. For the open test system, quantifications of creatinine at three levels were not interfered in the presence of dobutamine up to 20 μg/ml. In contrast, dobutamine exceeding 4 μg/ml falsely lowered the creatinine values, causing relative biases greater than 10%. Thus, for patients with dobutamine treatment, it seemed better to shift to open test system consisting of Shino‐Test assay kit, which was less perturbed. According to some reports, falsely low creatinine concentration could be determined in the presence of dobutamine or calcium dobesilate when using enzymatic methods.^31^, ^34^, ^35^ It is reported that dobutamine interferes stoichiometrically with all peroxidase‐based tests by being rapidly oxidized by peroxide in the presence of peroxidase, thus depleting the peroxide necessary to generate chromophore.^36^, ^37^ Guo et al.^31^ hypothesize that calcium dobesilate, as a hydroquinone ring, may increase the consumption of the hydrogen peroxide produced during the reaction to consequently negatively interfere the creatinine determination, or calcium dobesilate interferes with chromophore formation or influence the stability of chromophore generated. Of note, our study comprehensively evaluated analytical performances of two test systems that constituted creatinine assay kits from different manufacturers. The two test systems were comparable to each other, with good precision, accuracy, and linearity, although the open test system displayed stronger anti‐interference performance in the interference experiment. In conclusion, our study provides referable opinions for clinical laboratory selection. Clinicians and laboratory professionals should be mindful of potential interference caused by specified medications such as calcium dobesilate and dobutamine because an inaccurate creatinine result may lead to serious untoward consequences.

## Data Availability

The data that support the findings of this study are available from the corresponding author upon reasonable request.
